# Supplementary material for: Dissolved-Cl2 triggered redox reaction enables high-performance perovskite solar cells
Source: Nat Commun. 2023 Jun 22;14:3738. doi: 10.1038/s41467-023-39260-4 (PMC10287705; doi:10.1038/s41467-023-39260-4)
Supplement: Supplementary file 1 — Supplementary Information [file 41467_2023_39260_MOESM1_ESM.pdf]

Supplementary Information

Dissolved-Cl_2 Triggered Redox Reaction Enables High-performance Perovskite Solar Cells

Yujie Luo[1,†], Kaikai Liu[1,†], Liu Yang[1], Wenjing Feng,[1] Lingfang Zheng,[1] Lina Shen,[1] Yongbin Jin,[1] Zheng Fang,[1] Peiquan Song,[1] Wanjia Tian,[1] Peng Xu,[1] Yuqing Li,[1] Chengbo Tian[1], Liqiang Xie[1,*] & Zhanhua Wei[1,*]

[1] Xiamen Key Laboratory of Optoelectronic Materials and Advanced Manufacturing, Institute of Luminescent Materials and Information Displays, College of Materials Science and Engineering, Huaqiao University, Xiamen 361021, P.R. China

[†] These authors contributed equally to this work.

E-mails: lqxie@hqu.edu.cn; weizhanhua@hqu.edu.cn

This Supplementary Information includes:

Supplementary Figures 1 to 30

Supplementary Tables 1

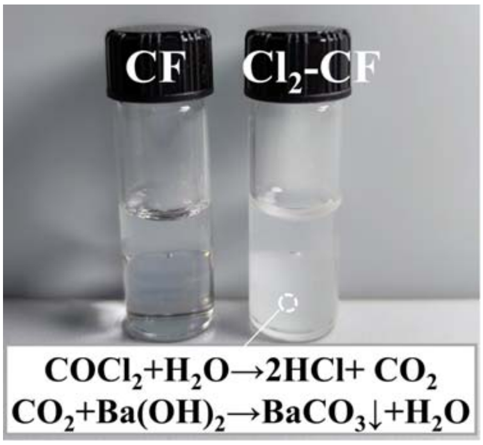

Supplementary Fig. 1 | Detecting the presence of CO_2 in Cl_2-CF using $Ba(OH)_2$. Test of CO_2 in Cl_2-CF using saturated barium hydroxide aqueous solution. When $Ba(OH)_2$ was added into Cl_2-CF and CF, a white precipitate of barium carbonate ($BaCO_3$) was generated in Cl_2-CF. In contrast, no obvious change was observed in CF.

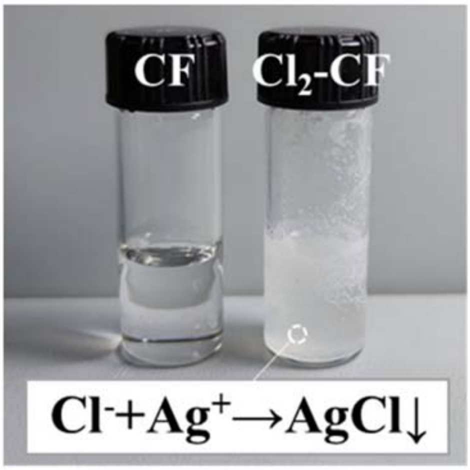

Supplementary Fig. 2 | Detecting the presence of Cl^- in Cl_2-CF using silver nitrate aqueous solution. Test of Cl^- in Cl_2-CF using silver nitrate aqueous solution. When silver nitrate was added to Cl_2-CF, it became turbid immediately.

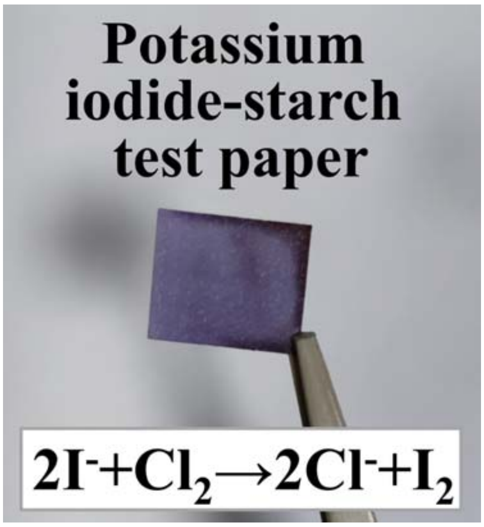

Supplementary Fig. 3 | Detecting the presence of Cl_2 in Cl_2-CF using wet starch potassium iodide test paper. The wet starch potassium iodide test paper was placed above the Cl_2-CF solvent and turned blue, proving the existence of Cl_2 in Cl_2-CF.

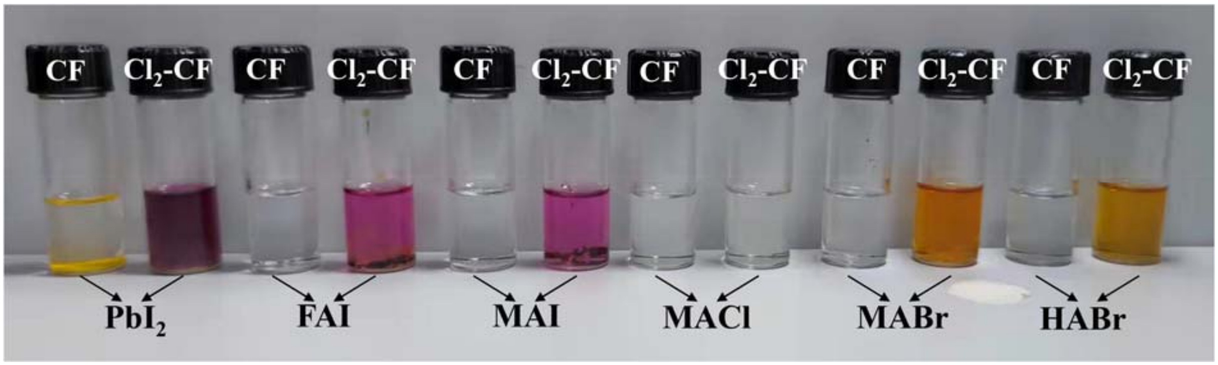

Supplementary Fig. 4 | Optical images of perovskite precursor compounds dissolved in Cl_2-CF. Photographs of perovskite precursor compounds dissolved in CF and Cl_2-CF.

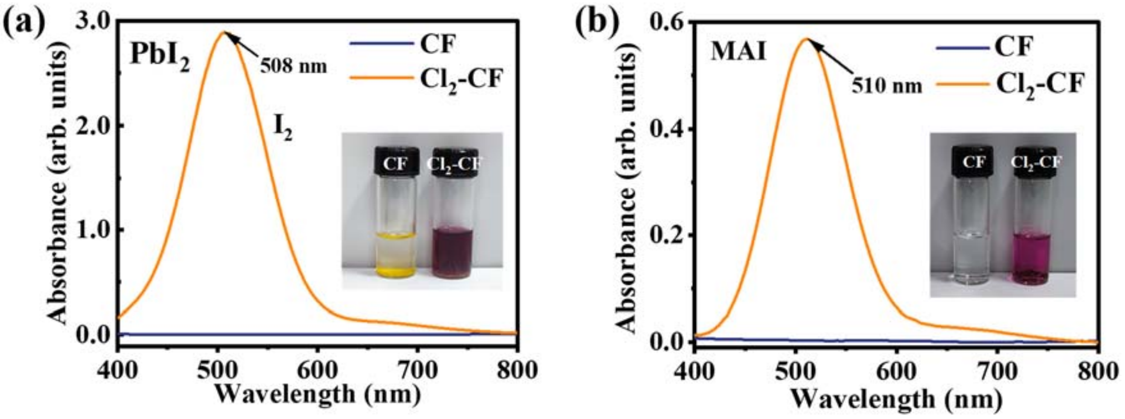

Supplementary Fig.5 | UV-vis absorption spectra of perovskite precursor compounds dissolved in CF and Cl_2-CF. UV-vis absorption spectra of (a) PbI_2 and (b) MAI dissolved in CF and Cl_2-CF.

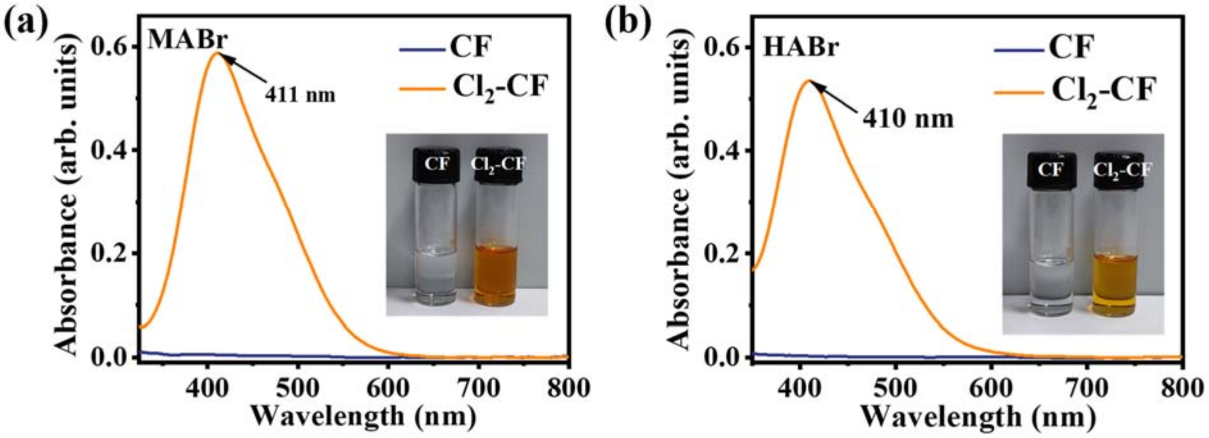

Supplementary Fig. 6 | UV-vis absorption spectra of perovskite precursor compounds dissolved in CF and Cl_2-CF. UV-vis spectra of (a) MABr and (b) HABr dissolved in CF and Cl_2-CF.

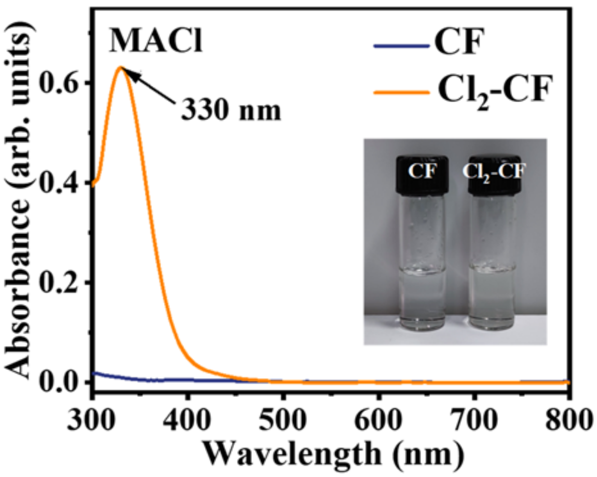

Supplementary Fig. 7 | UV-vis absorption spectra of perovskite MACl dissolved in CF and Cl_2-CF. The peak located at 330 nm indicates the presence of Cl_2.

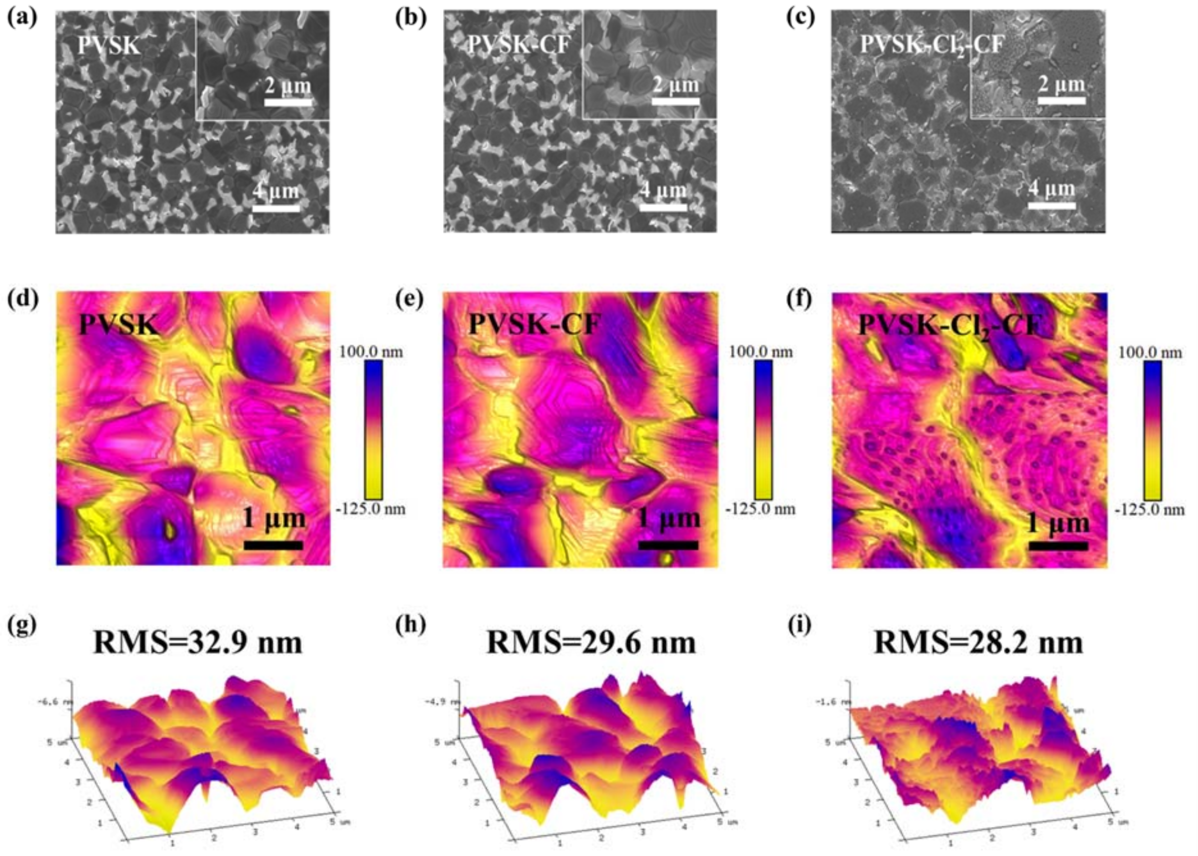

Supplementary Fig. 8 | Surface morphology of perovskite films. SEM images for (a) PVSK, (b) PVSK-CF, and (c) PVSK-Cl_2-CF. 2D AFM images of (d) PVSK, (e) PVSK-CF, and (f) PVSK-Cl_2-CF. The scan area of the AFM images is 5 × 5 μm^2. 3D AFM images of (g) PVSK, (h) PVSK-CF, and (i) PVSK-Cl_2-CF. The scan area of the 3D images is 5 × 5 μm^2.

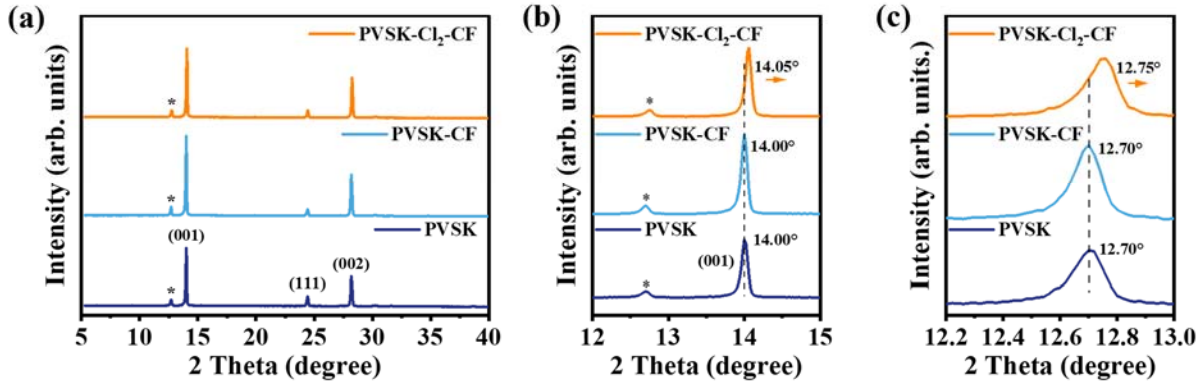

Supplementary Fig. 9 | XRD patterns of perovskite films. XRD patterns of PVSK, PVSK-CF, and PVSK-Cl_2-CF. (a) 5° to 40°, (b) 12° to 15°, (c) 12.2° to 13.0°.

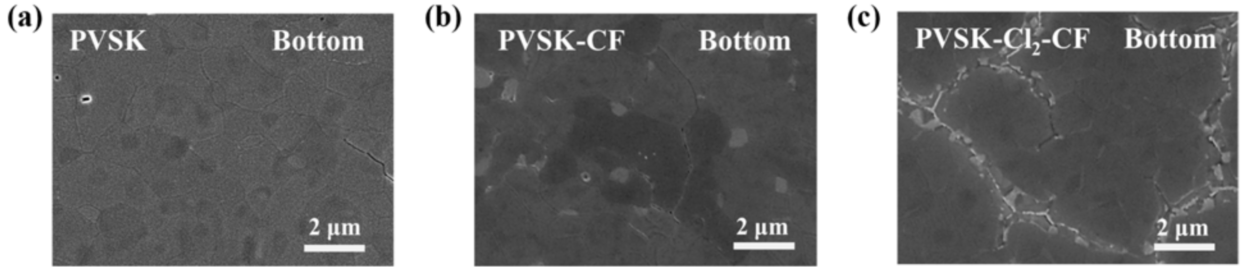

Supplementary Fig. 10 | Bottom-view SEM images of perovskite films. SEM images of the bottom surface of (a) PVSK, (b) PVSK-CF, and (c) PVSK-Cl_2-CF.

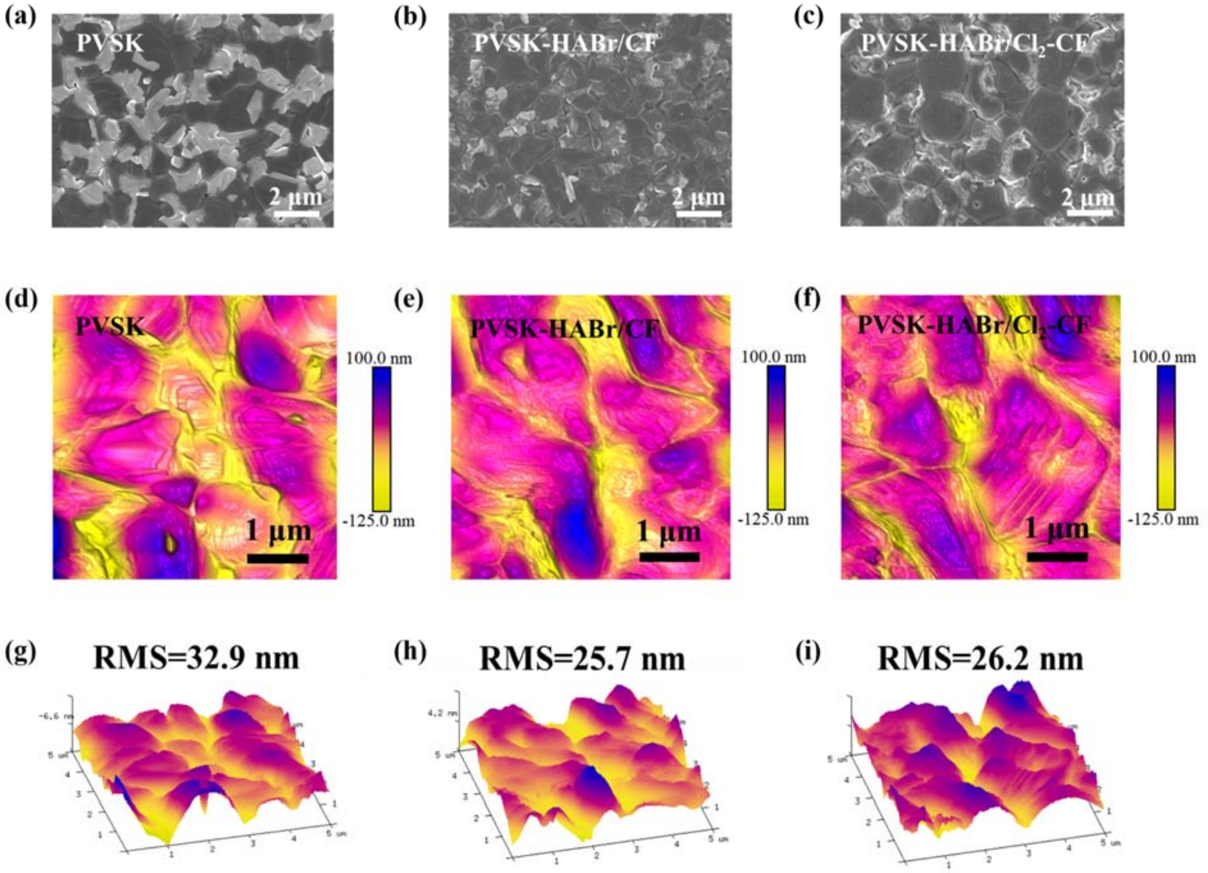

Supplementary Fig. 11 | Surface morphology of perovskite films. SEM images for (a) PVSK, (b) PVSK-HABr/CF, and (c) PVSK-HABr/Cl_2-CF. 2D AFM images of (d) PVSK, (e) PVSK-HABr/CF, and (f) PVSK-HABr/Cl_2-CF (scan area: 5 × 5 μm^2). 3D AFM images of (g) PVSK, (h) PVSK-HABr/CF, and (i) PVSK-HABr/Cl_2-CF (scan area: 5 × 5 μm^2).

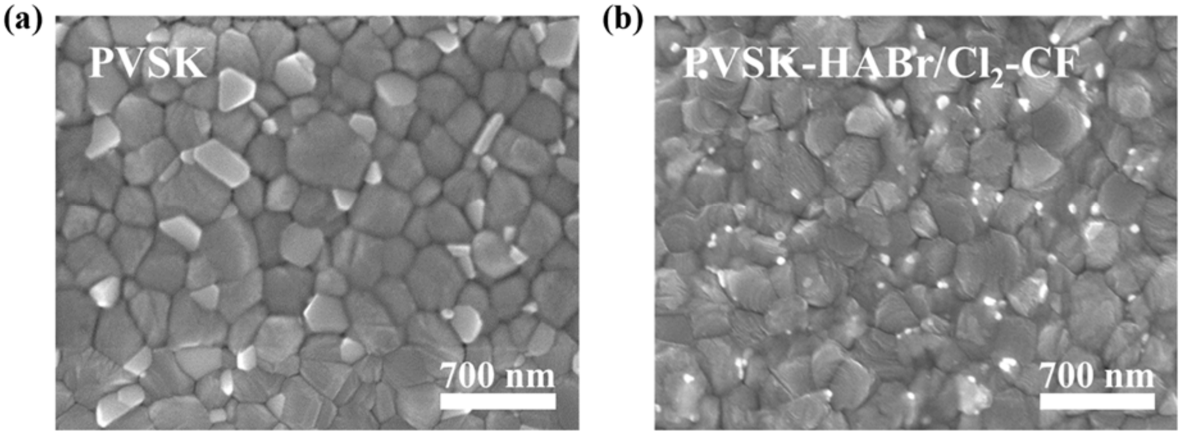

Supplementary Fig. 12 | Top-view SEM images of perovskite films. SEM images of perovskite films (a) without and (b) with HABr/Cl2-CF treatment. The perovskite films were fabricated by the one-step spin-coating method and showed almost a PbI2-free surface.

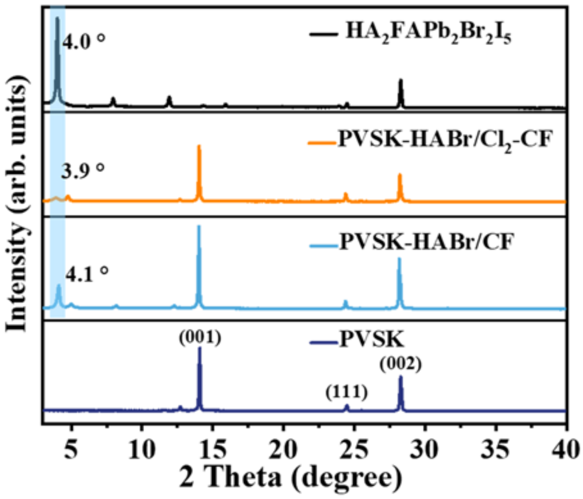

Supplementary Fig. 13 | XRD patterns of perovskite films. XRD patterns of PVSK, PVSK-HABr/CF, and PVSK-HABr/Cl2-CF and HA2FAPb2Br2I5.

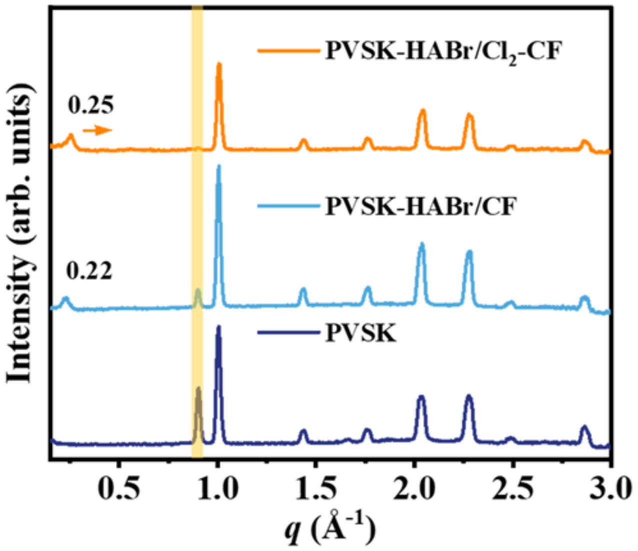

Supplementary Fig. 14 | Azimuthally integrated scattering patterns of the GIWAXS data. Azimuthally integrated GIWAXS characterization of PVSK, PVSK-HABr/CF, and PVSK-HABr/Cl_2-CF.

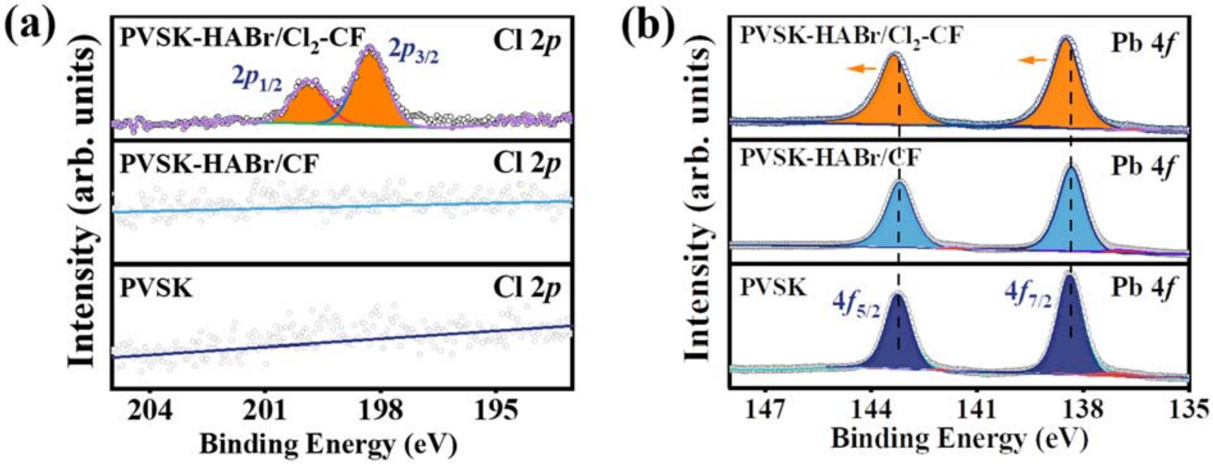

Supplementary Fig. 15 | XPS spectra of Cl 2*p* and Pb 4*f*. XPS for (a) Cl 2*p* and (b) Pb 4*f* spectra of PVSK, PVSK-HABr/CF, and PVSK-HABr/Cl_2-CF.

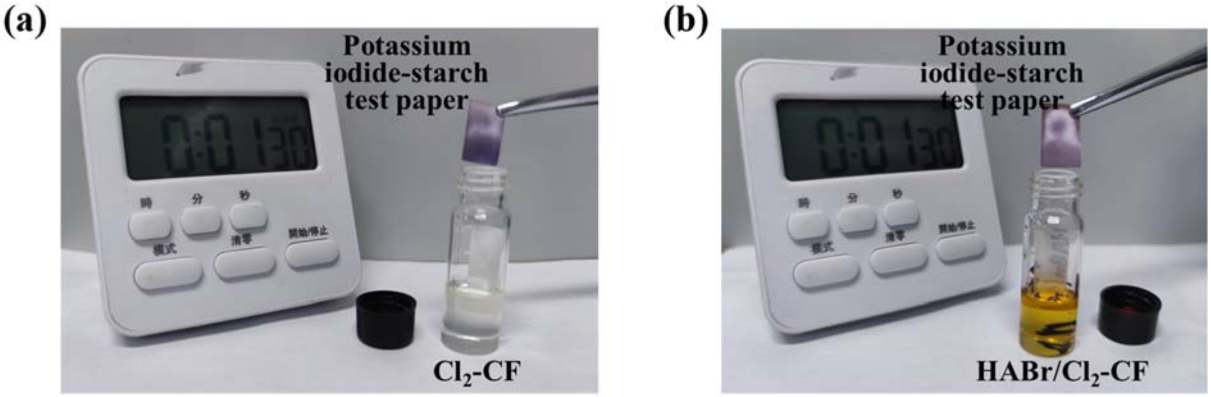

Supplementary Fig. 16 | Comparation of the state of Cl_2 in the Cl_2-CF solvent and the HABr/Cl_2-CF solution. (a) The wet starch potassium iodide test paper was placed above the Cl_2-CF solvent and turned blue, proving the existence of Cl_2 in Cl_2-CF. (b) The blue color proved the existence of Cl_2 in the HABr/Cl_2-CF solution although partial Cl_2 was consumed to oxidize the Br^- ions to Br_2.

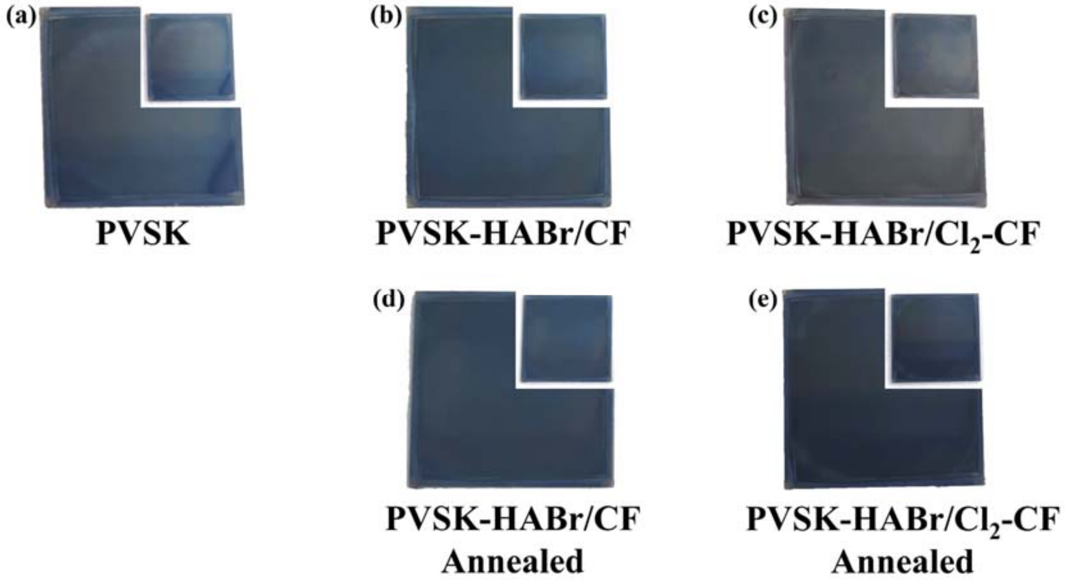

Supplementary Fig. 17 | Optical images of perovskite films in different treating stages. Photographs of (a) PVSK, (b) PVSK-HABr/CF, (c) PVSK-HABr/Cl_2-CF, (d) PVSK-HABr/CF Annealed, and (e) PVSK-HABr/Cl_2-CF Annealed.

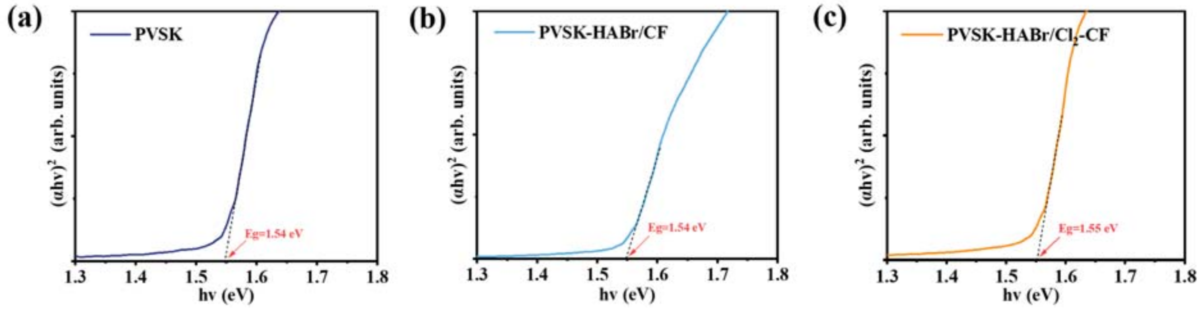

Supplementary Fig. 18 | Tuac plot of perovskite films. Tuac plot of (a) PVSK, (b) PVSK-HABr/CF, and (c) PVSK-HABr/Cl_2-CF.

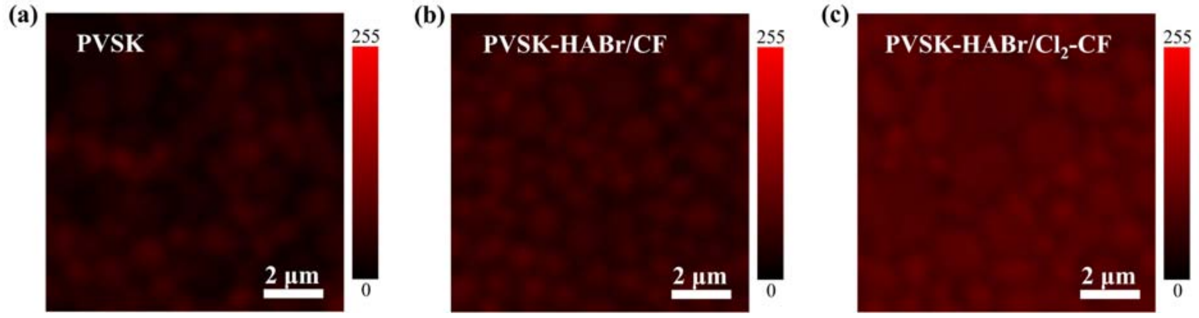

Supplementary Fig. 19 | 2D PL mapping images of perovskite films. 2D PL mapping images of (a) PVSK, (b) PVSK-HABr/CF, and (c) PVSK-HABr/Cl_2-CF.

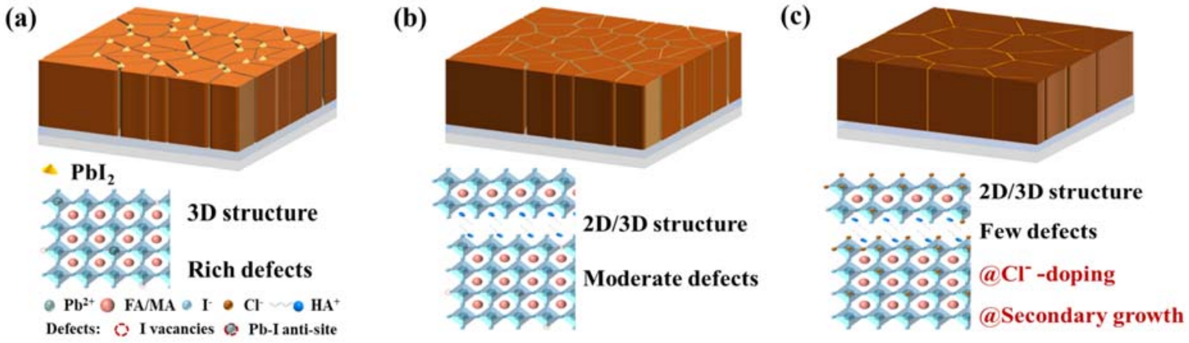

Supplementary Fig. 20 | Schematic illustration of the effect of post-treatment on perovskite films. Schematic illustration of the perovskite films for (a) PVSK, (b) PVSK-HABr/CF, and (c) PVSK-HABr/Cl_2-CF.

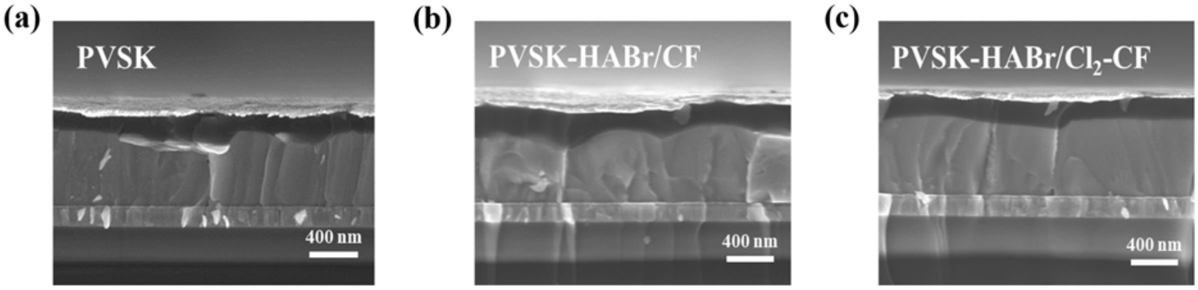

Supplementary Fig. 21 | Cross-sectional SEM images of PSCs. Cross-sectional SEM images of the (a) PVSK, (b) PVSK-HABr/CF, and (c) PVSK-HABr/Cl_2-CF based devices.

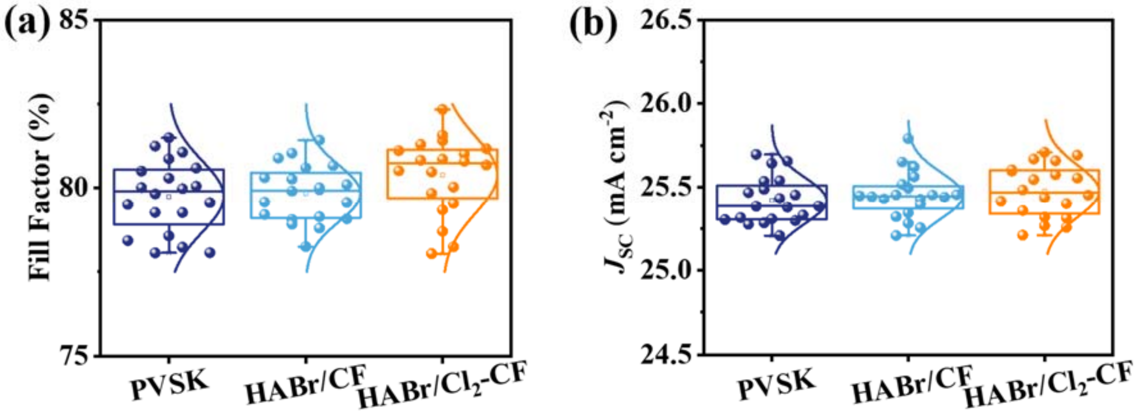

Supplementary Fig. 22 | Statistical FF and J_{SC} of perovskite solar cells. Statistics of (a) FF, and (b) J_{SC} of the devices based on PVSK, PVSK-HABr/CF, and PVSK-HABr/Cl_2-CF.

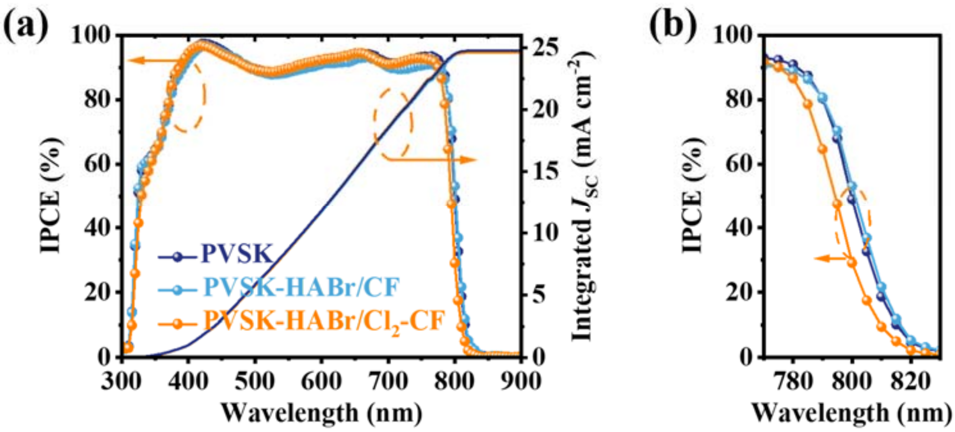

Supplementary Fig. 23 | IPCE spectra and the integrated J_{SC} of PSCs. IPCE spectra and the integrated J_{SC} of the PVSK, PVSK-HABr/CF, and PVSK-HABr/Cl_2-CF-based devices. (a) 300 to 900 nm, (b) 770 to 830 nm.

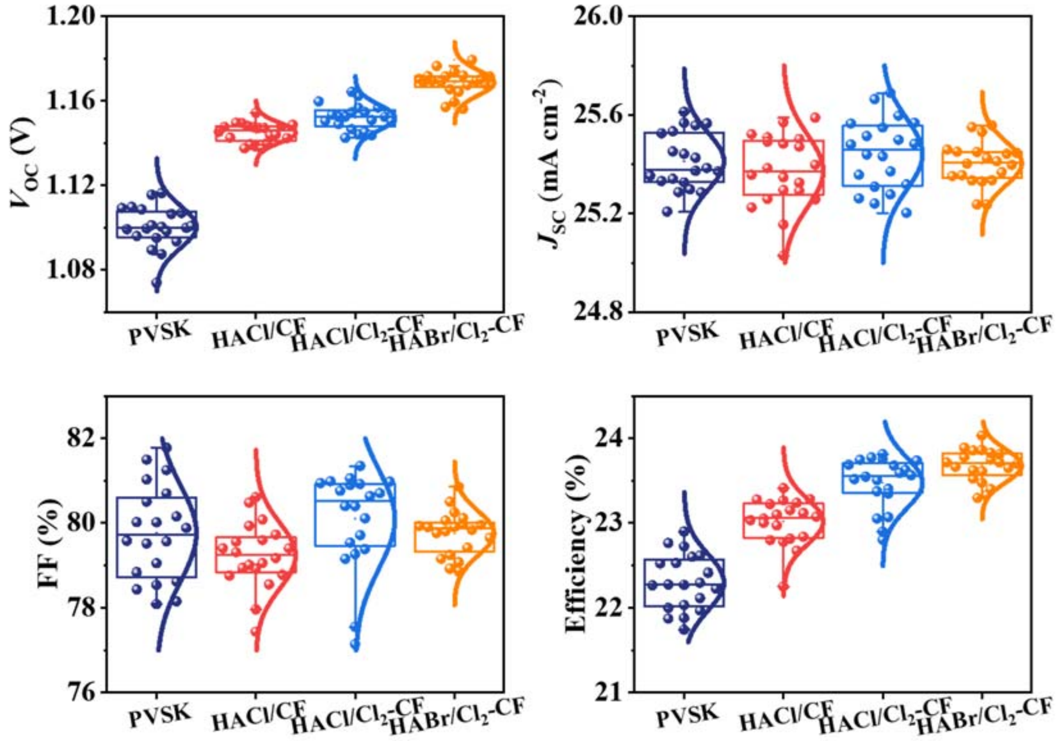

Supplementary Fig. 24 | Photovoltaic parameters of PSCs. Photovoltaic parameters of the PVSK, HACl/CF-treated, HACl/Cl_2-CF-treated, and PVSK-HABr/Cl_2-CF devices.

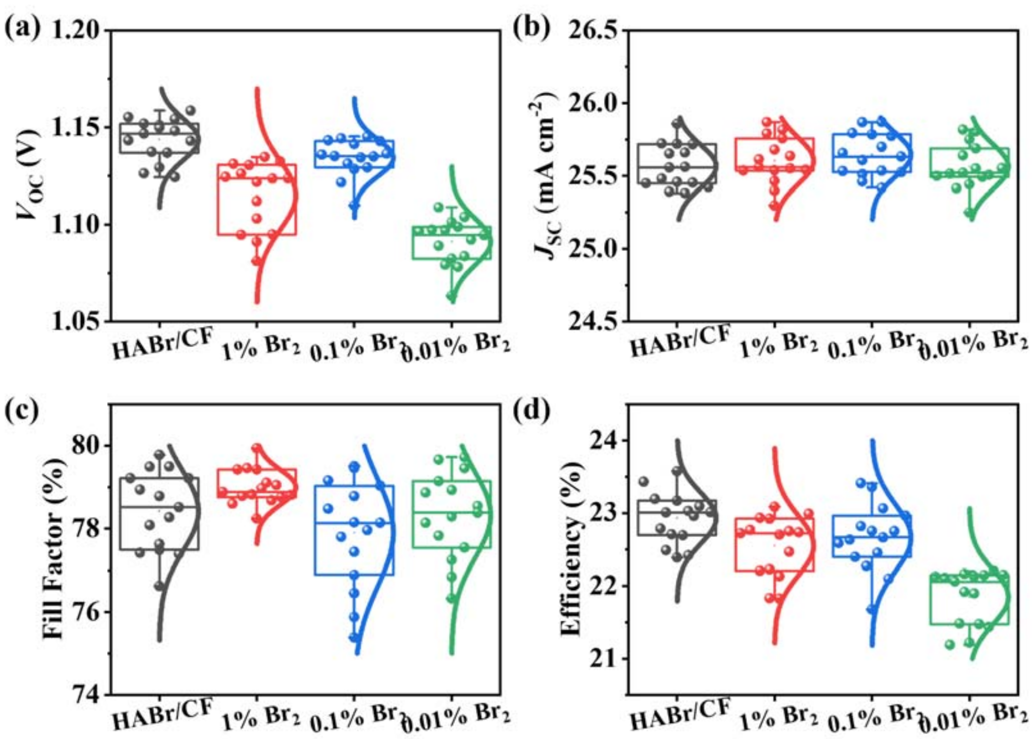

Supplementary Fig. 25 | Photovoltaic parameters of PSCs. Photovoltaic parameters of the devices post-treated by HABr/CF containing different ratios of Br_2 as additives. (a) V_{OC}, (b) J_{SC}, (c) fill factor and (d) efficiency.

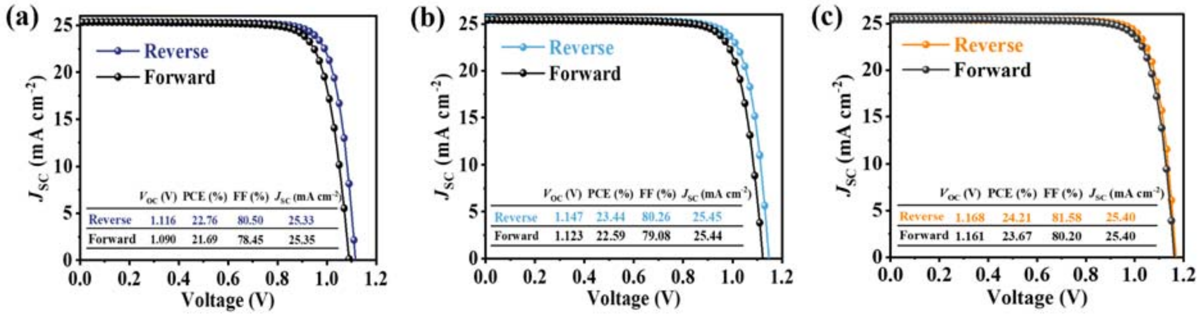

Supplementary Fig. 26 | *J-V* curves and hysteresis of PSCs. *J-V* curves and hysteresis of the (a) PVSK, (b) PVSK-HABr/CF, and (c) PVSK-HABr/Cl_2-CF based devices.

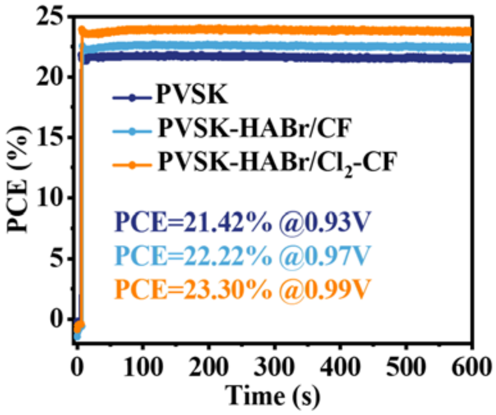

Supplementary Fig. 27 | Steady-state power output at the maximum power point of PSCs. Steady-state power output at the maximum power point of PVSK, PVSK-HABr/CF, and PVSK-HABr/Cl_2-CF-based devices.

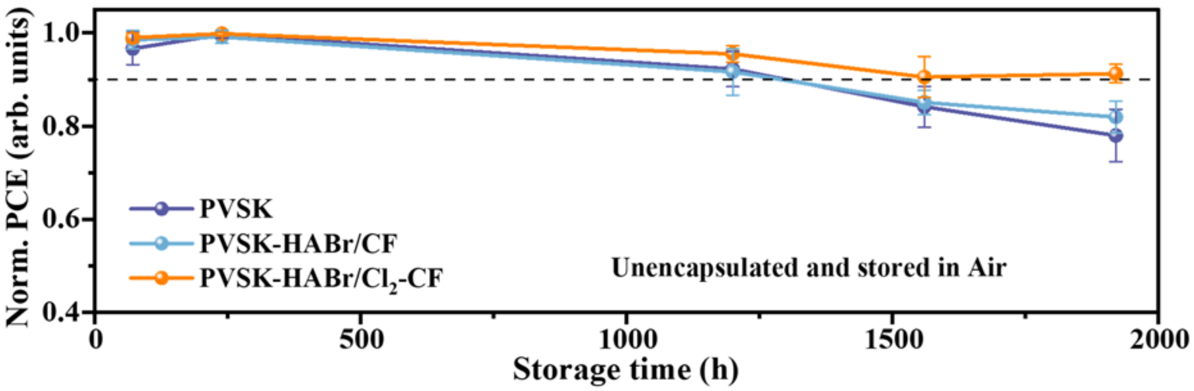

Supplementary Fig. 28 | Ambient stability of perovskite solar cells. Ambient stability of the PVSK, PVSK-HABr/CF, and PVSK-HABr/Cl2-CF-based devices. The devices were stored in the air with a relative humidity of ~10% and a temperature of ~25 °C. All the error bars represent the standard deviation for 8 devices. ‘Normalized’ is denoted as ‘Norm’.

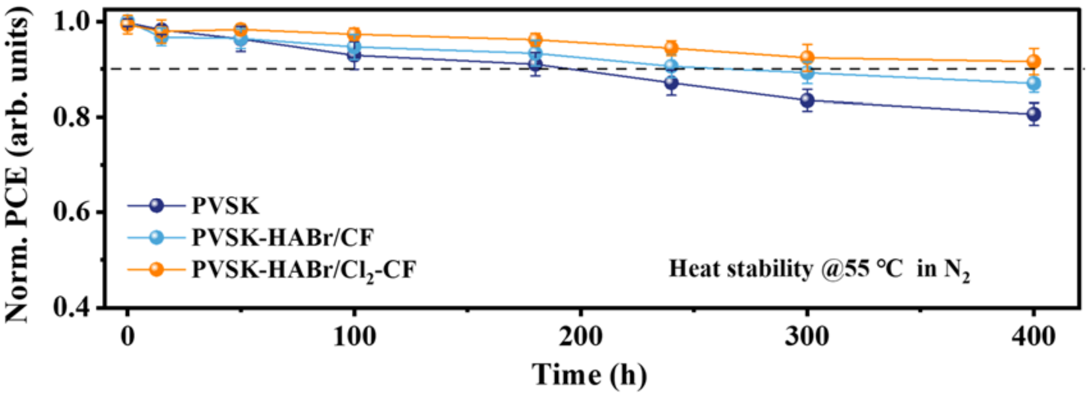

Supplementary Fig. 29 | Thermal stability (55°C) of perovskite solar cells. Thermal stability (55 °C) of the PVSK, PVSK-HABr/CF, and PVSK-HABr/Cl2-CF based devices. All the error bars represent the standard deviation for 8 devices. ‘Normalized’ is denoted as ‘Norm’.

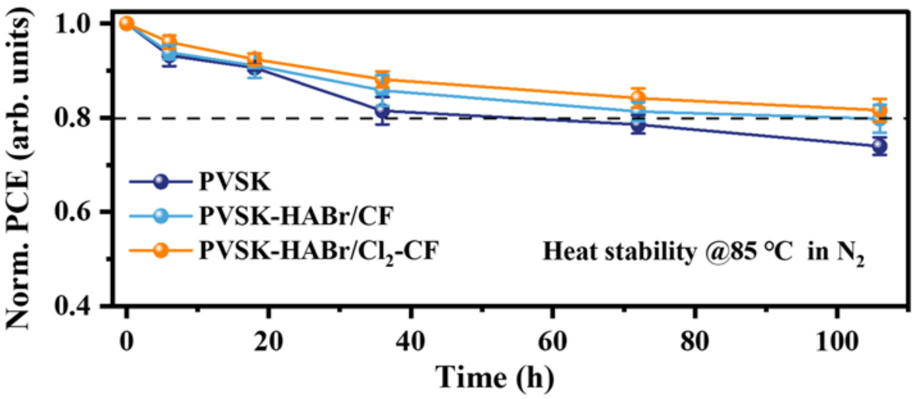

Supplementary Fig. 30 | Thermal stability (85 °C) of perovskite solar cells. Thermal stability (85 °C) of the PVSK, PVSK-HABr/CF, and PVSK-HABr/Cl_2-CF based devices. All the error bars represent the standard deviation for 5 devices. 'Normalized' is denoted as 'Norm'.

Supplementary Table S1 | Fitted parameters of TRPL results of perovskite films. Fitted parameters of TRPL results of PVSK, PVSK-HABr/CF, and PVSK-HABr/Cl_2-CF perovskite film.

	τ_1 (ns)	τ_2 (ns)	A_1	A_2	τ_{avg} (ns)
PVSK	188.8	901.3	498.7	1009.7	834.6
PVSK-HABr/CF	389.6	1176.8	377.9	880.0	1078.9
PVSK-HABr/Cl_2-CF	342.8	1712.4	238.2	913.8	1644.5
